# Activity level, kinesiophobia, and sports injury-related anxiety in professional athletes after anterior cruciate ligament reconstruction

**DOI:** 10.3389/fpsyg.2026.1830247

**Published:** 2026-07-27

**Authors:** Kadir Tiryaki, Miray Baser, Fatih Özden, Erkan Bingöl, Mehmet Özkeskin, Duygu Yaralı Bingöl, Atakan Çağlayan, İbrahim Çağrı Şanlı, Ahmet İmerci, Zübeyir Sarı

**Affiliations:** 1 Department of Physical Education and Sports, Faculty of Sport Sciences, Düzce University, Düzce, Türkiye; 2 Department of Physiotherapy and Rehabilitation, Institute of Health Sciences, İzmir Democracy University, İzmir, Türkiye; 3 Department of Health Care Services, Köyceğiz Vocational School of Health Services, Muğla Sıtkı Koçman University, Muğla, Türkiye; 4 Department of Property Protection and Security, Köyceğiz Vocational School, Muğla Sıtkı Koçman University, Muğla, Türkiye; 5 Department of Physiotherapy and Rehabilitation, Faculty of Health Sciences, Ege University, İzmir, Türkiye; 6 Institute of Sports Sciences, Muğla Sıtkı Koçman University, Muğla, Türkiye; 7 Department of Exercise and Sport Sciences, Faculty of Sport Sciences, İstanbul Gedik University, İstanbul, Türkiye; 8 Department of Orthopedics and Traumatology, Faculty of Medicine, Muğla Sıtkı Koçman University, Muğla, Türkiye; 9 Department of Physiotherapy and Rehabilitation, Faculty of Health Sciences, Marmara University, İstanbul, Türkiye

**Keywords:** anterior cruciate ligament reconstruction, kinesiophobia, return to sport, Sports Injury Anxiety, Tegner Activity Scale

## Abstract

**Introduction:**

This study aimed to examine the associations between anterior cruciate ligament reconstruction (ACL-R) status, activity level, kinesiophobia, and sports injury-related anxiety in professional athletes.

**Methods:**

This cross-sectional observational study included 115 athletes post-ACL-R and 153 age- and sex-matched healthy athletes. All participants completed the Tegner Activity Scale, Sports Injury Anxiety Scale, and Tampa Scale of Kinesiophobia–Short Version.

**Results:**

Athletes in the ACL-R group demonstrated significantly lower activity levels compared with controls (6.50 ± 2.63 vs. 8.12 ± 2.30, *p* < 0.001). No significant between-group differences were observed in sports injury-related anxiety (53.94 ± 13.08 vs. 52.77 ± 12.67, *p* = 0.643) or kinesiophobia (18.13 ± 3.01 vs. 18.05 ± 2.82, *p* = 0.990). Kinesiophobia and sports injury-related anxiety were significantly positively correlated in both the ACL-R group (*r* = 0.285, *p* = 0.002) and the control group (*r* = 0.365, *p* < 0.001). Tegner activity level was not significantly correlated with psychological variables in either group. In the ACL-R group, age, surgery year, and return-to-sport time were significant predictors of activity level, whereas sex, injury year, and psychological variables were not significant predictors.

**Conclusion:**

Professional athletes following ACL-R demonstrated lower activity levels than healthy controls, while kinesiophobia and sports injury-related anxiety were comparable between groups. Psychological variables were not independently associated with activity level after adjustment for demographic and injury-related factors.

## 1 Introduction

The growing interest in competitive sports, recreational activities, and fitness has led to an increasing incidence of anterior cruciate ligament (ACL) injuries (Bram et al., 2021). ACL injuries affect approximately 3% of amateur athletes and up to 15% of elite athletes annually (Mayer et al., 2015). These injuries not only impair athletic performance but also predispose the knee to long-term degenerative changes, including meniscal and cartilage damage, if left inadequately treated (Monllau et al., 2023; Acosta and Gobbi, 2025).

For young, active individuals, anterior cruciate ligament reconstruction (ACL-R) is generally considered the gold standard. Its goals are to restore joint stability, prevent further structural damage, and enable a safe return to high-intensity activities such as jumping, cutting, and rotation (Zaffagnini et al., 2015). Following an ACL-R, postoperative rehabilitation is a difficult and drawn-out process that starts as soon as surgery is performed and lasts until the athlete is judged ready to resume sports. Early rehabilitation focuses on pain and swelling management, restoration of full knee extension, and normalization of gait, while later stages incorporate progressive neuromuscular training to improve strength, endurance, balance, and cardiovascular fitness.

While activity level is often evaluated using objective measures, such as isokinetic strength testing and jump performance assessments, these criteria alone may not fully predict a successful return to sport (Gokeler et al., 2022; Wilk et al., 2024). It is becoming more well-acknowledged that psychological variables, such as kinesiophobia, anxiety associated with injuries, and fear of re-injury, are important predictors of healing and return to sport (Hsu et al., 2017; Baez et al., 2020). Several instruments are available to measure these variables. Still, their use in standard clinical practice remains limited, and there is insufficient data on the psychological effects of ACL-R, particularly among professional athletes (Huang et al., 2019).

This study aimed to examine the associations of ACL-R with activity level, kinesiophobia, and sports injury-related anxiety in professional athletes and to compare these outcomes with those of healthy control athletes. This approach provides a comprehensive understanding of both the physical and psychological dimensions of recovery, offering valuable insights to guide individualized rehabilitation and return-to-sport strategies.

## 2 Materials and methods

This study employed a quantitative, descriptive, cross-sectional design and was conducted online over a three-month period using Google Forms. Athletes who had undergone anterior cruciate ligament reconstruction (ACL-R) and were being followed by the Department of Orthopedics and Traumatology at Muğla Training and Research Hospital were invited to participate through Google Forms. The study population consisted of athletes aged 18–30 years who were actively competing at the national level and had a Tegner Activity Scale score of ≥6.

The term “professional athlete” in this study refers to individuals actively participating in organized national-level competitions and engaged in structured training within their respective sport federations. It is acknowledged that the inclusion criterion of a Tegner Activity Scale score ≥ 6 encompasses a heterogeneous performance spectrum ranging from highly competitive recreational athletes to elite-level competitors. This operational definition was chosen to ensure inclusion of athletes with a minimum threshold of regular sport participation, although variability in competitive level should be considered when interpreting the findings.

Participants were recruited through multiple online channels, including sports clubs, athlete communication groups, team mailing lists, and social media platforms. Coaches and team staff assisted in distributing the survey link to eligible athletes. Due to the open online dissemination strategy, the exact number of athletes who were exposed to the survey invitation could not be precisely determined; therefore, a formal response rate could not be calculated. However, all respondents who completed the survey within the data collection period were screened for eligibility according to inclusion and exclusion criteria.

A total of 268 athletes were included in the study, comprising 115 athletes in the ACL-R group and 153 athletes in the control group.

Athletes who had undergone unilateral ACL-R at least 9 months prior to participation were assigned to the ACL-R group. The control group consisted of healthy athletes with no history of serious lower extremity injury. Participants in the control group were matched to those in the ACL-R group on age and sex via frequency matching. Age categories were defined in 5-year intervals, and the proportions of male and female athletes in the control group were adjusted to match the distribution observed in the ACL-R group. No randomization procedures were applied.

Exclusion criteria included a body mass index > 30 kg/m^2^, current participation in a structured post-surgical rehabilitation program (for the ACL-R group), duplicate or multiple submissions of the online survey, and incomplete questionnaire data. Participants who withdrew or were excluded from the study were not followed up.

### 2.1 Participant flow and missing data

A total of 287 athletes initially responded to the online survey. Nineteen participants were excluded due to incomplete questionnaire data or duplicate submissions, resulting in a final sample of 268 athletes. No missing data were present in the final dataset; therefore, no imputation methods were applied.

### 2.2 Data collection

Independent variables included ACL-R status, age, sex, sport discipline (participants reported only their primary sport), injured extremity, year of injury, year of surgery, time since surgery, and return-to-sport time (months). Dependent variables were activity level, kinesiophobia, and sports injury-related anxiety, which were assessed using validated self-report questionnaires.

### 2.3 Tegner Activity Scale

An 11-level scale assessing activity level during work and sports participation. Level 0 represents complete disability due to injury, whereas level 11 corresponds to national-level competition. It is a reliable and valid tool for classifying activity level following ACL-R (Tegner and Lysholm, 1985).

### 2.4 Sports Injury Anxiety Scale (SIAS)

A 21-item, five-point Likert scale assessing anxiety related to sports injuries across seven subdomains (e.g., fear of reinjury, social support loss, pain). Higher scores indicate greater sports injury-related anxiety. The scale has been validated for Turkish athletes (Caz et al., 2019).

### 2.5 Tampa Scale for Kinesiophobia—short version (TSK-6)

It is a six-item short version of the 17-item form that measures fear of movement or reinjury associated with musculoskeletal disorders. Items are scored on a Likert-type scale, with higher total scores indicating greater kinesiophobia (Miller et al., 1991; Yilmaz et al., 2011). The TSK-6 was selected instead of the full 17-item version to reduce respondent burden in an online survey format and improve completion rates among professional athletes. The short version has demonstrated acceptable validity and reliability in musculoskeletal populations, including athletic samples. However, it is acknowledged that the abbreviated form may have reduced sensitivity to detect subtle variations in kinesiophobia within high-performance populations, which should be considered when interpreting null findings.

### 2.6 Statistical analysis

Statistical analyses were performed using SPSS version 27. Continuous variables are presented as mean ± standard deviation (SD), and categorical variables as frequencies and percentages [*n* (%)]. Normality of continuous variables was assessed using the Shapiro–Wilk test. Because several variables, including age and Tegner Activity Scale scores, did not meet normality assumptions, non-parametric statistical tests were applied as appropriate. Between-group comparisons were conducted using the Mann–Whitney U test for continuous variables and the chi-square test for categorical variables. Although non-parametric tests were used for inferential analyses, descriptive statistics are presented as mean ± SD to facilitate interpretation and comparison with previous literature. Correlation analyses between Tegner Activity Scale, TSK-6, and SIAS scores were performed separately for the ACL-R and control groups using Spearman’s rank correlation, with adjustment for age and sex. Correlation coefficients (r) and corresponding *p*-values are reported. Statistical significance was defined as *p* < 0.05. Hierarchical multiple linear regression analysis was performed within the ACL-R group to identify independent predictors of activity level (Tegner Activity Scale score). Demographic variables were entered in the first block, injury- and surgery-related variables in the second block, and psychological variables in the third block. Regression assumptions, including normality of residuals, linearity, homoscedasticity, independence of errors, and multicollinearity, were assessed prior to conducting analyses.

## 3 Results

The study included 115 athletes in the ACL-R group and 153 athletes in the control group. The mean age was 23.05 ± 3.67 years in the ACL-R group and 22.93 ± 3.27 years in the control group, with no significant difference between groups (*p* = 0.931). Sex distribution was also similar between groups (*p* = 0.476), with females comprising 33.0% of the ACL-R group and 37.3% of the control group. Regarding sport discipline, most participants in the ACL-R group were involved in team sports (90.4%), followed by individual sports (6.1%), fitness/training (2.6%), and combat/martial arts (0.9%). In the control group, 79.1% participated in team sports, 16.3% in fitness/training, and 4.6% in individual sports, while none reported participation in combat or martial arts.

In the ACL-R group, the mean year of injury was 2021.41 ± 3.45, and the mean year of surgery was 2022.11 ± 3.24. The dominant side was right in 69.6% and left in 30.4% of participants, whereas the injured side distribution was 55.7% right and 44.3% left. The mean return-to-sport time was 7.55 ± 4.11 months.

No significant between-group differences were observed in psychological outcomes, including SIAS and TSK-6 scores. The mean SIAS score was 53.94 ± 13.08 in the ACL-R group and 52.77 ± 12.67 in the control group (*p* = 0.643). Similarly, TSK-6 scores were comparable between groups (18.13 ± 3.01 vs. 18.05 ± 2.82; *p* = 0.990).

In contrast, activity level differed significantly between groups. Specifically, the Tegner Activity Level Score was significantly lower in the ACL-R group compared with the control group (6.50 ± 2.63 vs. 8.12 ± 2.30; *p* < 0.001) (Table 1).

**TABLE 1:** Demographic, clinical, and sport-related characteristics of participants in the ACL-R and control groups.

Variable	ACL-R group (*n* = 115)	Control group (*n* = 153)	*P*
Age (years), mean ± SD	23.05 ± 3.67	22.93 ± 3.27	0.931
Sex, *n* (%)			0.476
Female	38 (33.0%)	57 (37.3%)	–
Male	77 (67.0%)	96 (62.7%)	–
Sport discipline, n (%)			
Team sports†	104 (90.4%)	121 (79.1%)	–
Individual sports‡	7 (6.1%)	7 (4.6%)	–
Fitness/training	3 (2.6%)	25 (16.3%)	–
Combat sports§	1 (0.9%)	–	–
Year of injury, mean ± SD	2021.41 ± 3.45	–	–
Year of surgery, mean ± SD	2022.11 ± 3.24	–	–
Dominant side, *n* (%)			–
Right	80 (69.6%)	98 (64.1%)	–
Left	35 (30.4%)	55 (35.9%)	–
Injured side, n (%)			
Right	64 (55.7%)	–	–
Left	51 (44.3%)	–	–
Return-to-sport time (months), mean ± SD	7.55 ± 4.11	–	–
Sports Injury Anxiety Scale, mean ± SD	53.94 ± 13.08	52.77 ± 12.67	0.643
Tampa Scale for Kinesiophobia–6, mean ± SD	18.13 ± 3.01	18.05 ± 2.82	0.990
Tegner Activity Level Score, mean ± SD	6.50 ± 2.63	8.12 ± 2.30	<0.001*

†Soccer, basketball, volleyball, handball, American football. ‡Swimming, tennis, running, trekking, trampoline. §Kickboxing, Muay Thai, karate, judo, taekwondo, fencing, capoeira. ACL-R, anterior cruciate ligament reconstruction; SD, standard deviation.

*Statistically significant at *p* < 0.05. *P*-values are not reported for sport discipline due to descriptive presentation. *P*-values for categorical variables were calculated using the chi-square test.

Participants in the ACL-R group were stratified according to time since surgery into three categories: ≤ 2 years (46.1%), 3–5 years (34.8%), and ≥6 years (19.1%). This distribution indicates that nearly half of the sample was in the early post-surgical period. A Kruskal–Wallis test revealed a significant difference in Tegner Activity Level Score across groups [H(2) = 10.661, *p* = 0.005]. *Post hoc* pairwise comparisons using Mann–Whitney U tests with Bonferroni correction (α = 0.017) showed a significant difference between the ≤2 and 3–5 years groups (U = 685.0, *p* = 0.003). However, no significant differences were observed between the ≤2 and ≥6 years groups (U = 383.0, *p* = 0.019) or between the 3–5 and ≥6 years groups (U = 432.5, *p* = 0.911) after adjustment for multiple comparisons. In contrast, no significant differences were found for SIAS or TSK-6 scores across time-since-surgery groups (SIAS: *p* = 0.495; TSK-6: *p* = 0.837).

Correlation analyses adjusted for age and sex were conducted separately for the ACL-R and control groups. In the ACL-R group, Tegner scores were not significantly correlated with TSK-6 (*r* = 0.070, *p* = 0.461) or SIAS (*r* = −0.061, *p* = 0.519). In contrast, TSK-6 and SIAS showed a significant positive correlation (*r* = 0.285, *p* = 0.002). Similarly, in the control group, Tegner scores were not significantly correlated with TSK-6 (*r* = 0.113, *p* = 0.169) or SIAS (*r* = 0.059, *p* = 0.475). However, a significant positive correlation between TSK-6 and SIAS was also observed (*r* = 0.365, *p* < 0.001) in the control group (Table 2).

**TABLE 2:** Correlation between tegner activity level score, TSK-6, and SIAS in ACL-R and control groups (adjusted for age and sex).

Group	Variables	Tegner Activity Level Score	Tampa Scale for Kinesiophobia	Sports Injury Anxiety Scale
ACL-R	Tegner Activity Level Score	1	–	–
	Tampa Scale for Kinesiophobia	0.070 (*p* = 0.461)	1	–
	Sports Injury Anxiety Scale	−0.061 (*p* = 0.519)	0.285 (*p* = 0.002)*	1
Control	Tegner Activity Level Score	–	–	–
	Tampa Scale for Kinesiophobia	0.113 (*p* = 0.169)	1	–
	Sports Injury Anxiety Scale	0.059 (*p* = 0.475)	0.365 (*p* < 0.001)*	1

ACL-R, Anterior Cruciate Ligament Reconstruction; SIAS, Sports Injury Anxiety Scale; TSK-6, Tampa Scale for Kinesiophobia 6-item. Partial correlation coefficients controlling for age and sex.

*P < 0.05.

As shown in Table 3, hierarchical regression analysis indicated that in Model 1, age significantly predicted activity level (β = −0.331, t = −2.894, *p* = 0.005), whereas sex was not a significant predictor (β = 0.039, t = 0.343, *p* = 0.733). Model 1 explained 9.8% of the variance in activity level (R^2^ = 0.098, *p* = 0.009).

**TABLE 3:** Hierarchical regression analysis predicting activity level (Tegner Activity Level Score) in the ACL-R group.

Model	Predictor	β	t	*P*
Model 1				
R^2^ = 0.098, Adj. R^2^ = 0.078	Age	−0.331	−2.894	0.005
	Sex	0.039	0.343	0.733
Model 2				
R^2^ = 0.205, Adj. R^2^ = 0.160	Age	−0.310	−2.808	0.006
	Surgery year	−0.444	−2.392	0.019
	Return-to-sport time	0.212	2.222	0.029
	Injury year	0.325	1.743	0.085
Model 3				
R^2^ = 0.217, Adj. R^2^ = 0.154	SIAS	−0.097	−1.004	0.318
	TSK-6	0.076	0.774	0.441

ΔR^2^ Model 1, 0.098 (*p* = 0.009); ΔR^2^ Model 2, 0.107 (*p* = 0.010); ΔR^2^ Model 3, 0.013 (*p* = 0.501). ΔR^2^: Change in R-squared (explained variance added by each block); SIAS, Sports Injury Anxiety Scale; TSK-6, Tampa Scale for Kinesiophobia–Short Version (6-item); β, standardized regression coefficient. The regression analysis was conducted exclusively within the ACL-R group. Variance inflation factor (VIF) values were <5 for all predictors, indicating no multicollinearity. Regression assumptions (normality of residuals, linearity, homoscedasticity, and independence of errors) were examined and met.

In Model 2, the addition of injury- and surgery-related variables significantly improved model fit (ΔR^2^ = 0.107, *p* = 0.010), increasing explained variance to 20.5% (R^2^ = 0.205). Within this model, age (β = −0.310, *p* = 0.006), surgery year (β = −0.444, *p* = 0.019), and return-to-sport time (β = 0.212, *p* = 0.029) were significant predictors, whereas injury year was not (*p* = 0.085).

In Model 3, the addition of psychological variables (SIAS and TSK-6) did not significantly improve model fit (ΔR^2^ = 0.013, *p* = 0.501), with the final model explaining 21.7% of the variance in activity level (R^2^ = 0.217). Neither SIAS nor TSK-6 significantly predicted activity level beyond demographic and injury-related variables.

## 4 Discussion

### 4.1 Main findings

Anterior cruciate ligament injuries are common among athletes, and surgical repair is often required to restore knee stability and enable return to sport. In comparison to healthy controls, this study examined associations between ACL-R status and activity level, kinesiophobia, sports injury-related anxiety, performance anxiety, and fear of re-injury. A psychological factor that has been widely acknowledged to influence rehabilitation outcomes and return-to-sport readiness is kinesiophobia, which is the fear of movement due to the expectation of pain or reinjury. Sports injury-related anxiety includes worries about sustaining a new injury or exacerbating an existing one, and performance anxiety represents stress related to reaching pre-injury performance levels (Ambegaonkar et al., 2024). Fear of reinjury, closely related to both kinesiophobia and sports injury-related anxiety, has been shown to negatively impact both physical and psychological recovery after ACL-R (Hsu et al., 2017; Nedder et al., 2025).

Demographic characteristics (age and sex) were comparable between the ACL-R and control groups, suggesting that observed differences in outcomes are unlikely to be confounded by these variables. This strengthens the internal validity of between-group comparisons.

Sport participation patterns differed between groups, with a higher proportion of team sport athletes in the ACL-R group compared with controls, while controls more frequently participated in fitness/training activities. Given the higher biomechanical and neuromuscular demands of team sports involving cutting, pivoting, and contact movements, this imbalance may have influenced activity level outcomes and psychological responses (Barber-Westin and Noyes, 2020; Resch et al., 2025). Sport discipline was not included as a covariate in the main analyses and should therefore be considered when interpreting the findings.

Injury and surgical variables showed that the mean injury year was 2021.41 ± 3.45 and the mean surgery year was 2022.11 ± 3.24, with the dominant side being right in 69.6% of ACL athletes and the injured side right in 55.7%. The mean return-to-sport time was 7.55 ± 4.11 months, aligning with previously reported averages of 6–9 months following ACL-R (Harris et al., 2014; Randsborg et al., 2022). Other reports, however, suggest that the results of returning to sports following an ACL tear can vary substantially. Return-to-sport times of 12 months or more have been documented in systematic reviews, particularly among athletes with concurrent injuries or who require revision surgery (Wiggins et al., 2016). In addition, narrative reviews recommend delaying full sport participation up to 18–24 months to allow optimal graft maturation, neuromuscular recovery, and psychological readiness, including mitigation of fear of reinjury (Grindem et al., 2016). Such variability underscores that although the mean return-to-sport duration observed in our cohort aligns with typical averages, individualized rehabilitation strategies and careful assessment of both physical and psychological criteria remain essential for a safe and sustainable return to pre-injury sport levels.

Participants in the ACL-R group were stratified according to time since surgery (≤2, 3–5, ≥6 years). A significant difference in activity level was observed across groups, primarily driven by differences between the ≤2 and 3–5 subgroups. No significant differences were observed for psychological variables across time-since-surgery categories.

### 4.2 Psychological and functional outcomes

Activity level was associated with age, surgery year, and return-to-sport time; however, sex, injury year, and psychological variables were not significantly associated with activity level. These findings suggest that demographic and injury/surgery-related factors show stronger associations with activity level than psychological variables in this cohort.

Psychological outcomes demonstrated no significant differences between ACL-R athletes and controls in TSK-6 or SIAS scores. Partial correlation analyses showed a significant positive relationship between kinesiophobia and sports injury-related anxiety in both groups, whereas activity level was not significantly associated with either psychological measure. These findings suggest that while fear and anxiety are interrelated constructs, they may not directly determine sport participation or activity level once athletes have returned to training or competition (Ardern et al., 2014; Zaffagnini et al., 2015).

Therefore, psychological variables in this sample were not independently associated with activity level when demographic and injury-related factors were considered. The finding that more recent surgical year was associated with lower activity level warrants careful interpretation. This result may reflect the temporal proximity to surgery, where athletes with more recent operations are still in earlier phases of functional recovery and sport reintegration. In addition, surgical year is conceptually related to time since surgery, and partial overlap between these variables may introduce interpretive complexity. Therefore, this finding should be interpreted as reflecting recovery timing effects rather than a direct effect of calendar year of surgery.

The absence of significant differences in psychological variables between groups may be explained by survivorship bias, as athletes who successfully returned to sport may represent a subgroup with lower levels of fear and anxiety. Additionally, the use of the TSK-6 may have limited sensitivity to detect subtle differences in high-performance athletes, potentially contributing to floor effects. Overall, these findings may reflect both true psychological adaptation in successfully returned athletes and measurement limitations in elite sport populations.

### 4.3 Activity level differences

Activity level was significantly lower in ACL-R athletes compared with controls, indicating that full restoration of preinjury activity capacity may not yet be achieved following return to sport. This supports prior evidence that post-reconstruction performance and participation may remain impaired for a period, particularly in team sports (Kaplan and Witvrouw, 2019; Fort-Vanmeerhaeghe et al., 2022; Manojlovic et al., 2024).

From a physiotherapy perspective, these results highlight the importance of integrating psychological readiness assessment with objective functional evaluation during rehabilitation. Lower activity levels despite similar psychological profiles may reflect residual neuromuscular deficits, reduced performance capacity, sport-specific demands, and incomplete recovery of preinjury function. Therefore, rehabilitation should prioritize restoration of strength, neuromuscular control, and sport-specific performance to support safe return to sport.

### 4.4 Limitations

Although this study used validated psychosocial tools and included a well-matched control group, several limitations should be acknowledged. First, self-reported questionnaires were used instead of objective functional assessments; therefore, future studies combining psychological measures with performance-based and biomechanical evaluations may provide a more comprehensive understanding of recovery after ACL-R. Second, most participants were young professional athletes, which limits the generalizability of the findings to older individuals or recreational populations. No missing data were present in the final dataset, and all participants were included in the analyses. In addition, the difference in sport discipline distribution between groups may have acted as a potential confounding factor influencing activity level outcomes. The cross-sectional design precludes any inference regarding causal relationships between ACL-R status and the studied variables. Furthermore, the use of online recruitment may have introduced selection bias by favoring athletes with greater digital engagement and internet accessibility, thereby potentially limiting the representativeness of the sample. Finally, because the study included only Turkish athletes, caution is warranted when generalizing the findings to populations with different cultural backgrounds, healthcare systems, and rehabilitation protocols.

### 4.5 Conclusion

Professional athletes who undergo ACL-R demonstrate reduced activity levels even after returning to sport. Levels of kinesiophobia and sports injury-related anxiety are comparable to those of healthy peers. Activity level appears to be more strongly influenced by demographic and surgical factors than by psychological variables in this cohort. Rehabilitation should therefore prioritize the restoration of strength, neuromuscular control, and sport-specific performance to support a safe and successful return to sport.

## Data Availability

The raw data supporting the conclusions of this article will be made available by the authors, without undue reservation.
